# Aesthetic Image Statistics Vary with Artistic Genre

**DOI:** 10.3390/vision4010010

**Published:** 2020-02-01

**Authors:** George Mather

**Affiliations:** University of Lincoln, School of Psychology, Lincoln LN6 7AY, UK; gmather@lincoln.ac.uk

**Keywords:** image statistics, spectral slope, fractal dimension, entropy, aesthetics

## Abstract

Research to date has not found strong evidence for a universal link between any single low-level image statistic, such as fractal dimension or Fourier spectral slope, and aesthetic ratings of images in general. This study assessed whether different image statistics are important for artistic images containing different subjects and used partial least squares regression (PLSR) to identify the statistics that correlated most reliably with ratings. Fourier spectral slope, fractal dimension and Shannon entropy were estimated separately for paintings containing landscapes, people, still life, portraits, nudes, animals, buildings and abstracts. Separate analyses were performed on the luminance and colour information in the images. PLSR fits showed shared variance of up to 75% between image statistics and aesthetic ratings. The most important statistics and image planes varied across genres. Variation in statistics may reflect characteristic properties of the different neural sub-systems that process different types of image.

## 1. Introduction

In 1924, the artist Fernand Leger stated that “beauty is everywhere, in the arrangement of your pots and pans, on the white wall of your kitchen, more perhaps than in your eighteenth century salon or in the official museum”. Piet Mondrian stated, in 1937, that artists aim “at the direct creation of universal beauty”, thus agreeing with Leger’s idea that beauty is everywhere and in all things [1]. The question of whether ‘universal beauty’ exists has been debated by philosophers for centuries, but there seems little doubt today that certain objects are consistently rated as more beautiful than others [2,3,4], although the reasons for such consistency are still unclear. In recent years, vision scientists have tried to identify an objective property in artworks that signifies their universal beauty. In the context of visual art, this search has taken the form of a comparison between viewer ratings of the aesthetic pleasure evoked by paintings and objective, statistical measurements of certain low-level image properties. The question then becomes: is aesthetic pleasure linked to a particular universal statistical property of artworks? A large number of studies have investigated the relation between image statistics and aesthetic responses (e.g., [5,6,7]) and have found some relation between statistics and preferences, but only a relatively small number have specifically assessed the strength of correlations between image properties and viewer preferences across a range of artworks in order to test for universality. Mallon et al. [8] found shared variance of 3.3% to 7.7% between their various image statistics and subjective ratings. On the other hand, Forsythe et al. [9] found a stronger relationship between a combination of their image complexity statistics (GIF image size and fractal dimension) and beauty ratings (42% shared variance). However, a recent paper [10] found no more than 5.6% shared variance between their artwork statistics and ratings. When analyses were performed separately for artworks containing different subjects, shared variance increased, and was highest for flowers/vegetation (13%) and for buildings (25.3%). Other recent research has focused on identifying clusters of participants who share common statistical preferences [10,11,12]

The variable success of attempts to identify simple statistical measures of universal beauty is not surprising given that artworks vary along multiple visual dimensions and in terms of their semantic content: as well as containing luminance and colour variation at a range of spatial scales, artworks also often contain meaningful subject matter, such as a landscape, a human face or other biological forms. Given this complexity, beauty ratings may depend on a combination different image statistics rather than a single statistic, and the relevant combination of statistics may vary across subject matter. As a test of this conjecture, this study used partial least squares regression (PLSR) to investigate the relation between ratings of a range of different artwork genres and combinations of three image statistics. These statistics were computed because they are the most widely used measures in the literature on empirical aesthetics and visual processing, as indicated by the references cited above and below.

The following three statistics were applied separately to the achromatic and chromatic content of each image using the CIELAB image representation.

(1) Fractal Dimension (FD)

FD is based on fractal geometry and estimates a picture’s geometric complexity in terms of the extent to which it is fractured or broken up into a pattern that fills the two-dimensional picture space. FD has been proposed to play an important role in aesthetic ratings in several studies [5,6,7].

(2) Spectral Slope (SL)

SL is a measure of an image’s photometric properties. It estimates tonal variation in the lightness or chroma of a picture at many different spatial scales covering a spectrum from coarse scale to fine scale. SL summarises this variation in a ‘slope’ value that represents the relative preponderance of coarse and fine scale tonal variation [13,14,15].

(3) Entropy (EN)

EN measures the extent to which information in a picture varies unpredictably. Pictures in which tonal and colour values at any one location can be predicted very well by the values nearby are highly redundant and have EN values close to zero; images in which tonal and colour values are very unpredictable have high EN values. EN is thought to be an important factor in the efficiency with which images can be encoded [16,17].

In addition to correlating each of the statistics individually with aesthetic ratings, an important goal of the research was to use PLSR to find the optimal combination of statistics that explains the obtained ratings [18]. PLSR is similar to principal component analysis (PCA) in that it generates a component model based on linear combinations of predictor variables. However, rather than creating components to explain the variability only in a set of predictor variables, as PCA does, PLSR creates components that take into account both the predictor and the response variables. PLSR requires few assumptions and can be used when predictor variables are highly collinear (correlated). Following the application of PLSR, variable importance in projection (VIP) scores were calculated to estimate the importance of each predictor variable (i.e., image statistic) in the best-fitting model [19], as an aid to understanding which of the statistics best explain variance in ratings. For both correlations and PLSR fits, the percentage of variance explained was calculated as (r^2^ * 100) to provide a summary measure of the goodness-of-fit to ratings.

## 2. Materials and Methods

### 2.1. Images

Artistic images were drawn from a large set of images previously used in research on image statistics [20]. This set contained 476 figurative and abstract artworks dating from 1435 to 2008. Aesthetic ratings of these images are available in the JenAesthetics [21] and MART databases [22]. As in the previous work [20], the sample of images from the two databases was constrained so that each artwork in the image set was produced by a different artist. The MART database contains only abstract works, while the JenAesthetics database contains artworks depicting a range of subjects, tagged by the creators of the database according to a number of categories. These same categories were employed in the present analysis in order to maintain consistency and comparability with other research that utilises the JenAesthetics database and categories.

### 2.2. Subjective Ratings

The JenAesthetics database includes ratings for ‘beauty’ with data recorded on a 100-point scale. The MART database includes ratings for the ‘emotion’ evoked by each painting on a 7-point scale (1 representing a highly negative emotion and 7 representing a highly positive one). Assuming that the latter scores bear some relation to beauty, they were re-scaled onto a 100-point scale so that they could be combined with the JenAesthetics ratings.

### 2.3. Calculation of Image Statistics

Calculations were carried out in Matlab^®^, using the same algorithms as used previously [20]. Each image was cropped to the largest central square region and then re-scaled to 1024 × 1024 pixels using cubic interpolation (Matlab^®^ imresize function) so that statistics were not affected by variations in the pixel dimensions and the aspect ratios of different images. Each image was then converted to CIELAB colour space using the Matlab^®^ makecform (‘srgb2lab’) function. CIELAB colour space is commonly used in empirical aesthetics [23]. Statistics were calculated separately on the **L** (luminance), **a** (red-green), and **b** (blue-yellow) colour planes of each image as follows: 

*Spectral slope* (SL). A 2-D Fourier transform was computed using built-in Matlab^®^ functions, and the slope of the fitted line to the rotationally averaged amplitude spectrum on log-log co-ordinates was extracted. Natural images tend to have a spectral slope of about -1.2. Steeper slopes correspond to images with a greater preponderance of low spatial frequency content relative to high spatial frequency content.

*Fractal dimension* (FD). The fractal dimension of each image was calculated using a 3-D box-counting algorithm, in which image content is represented as a surface in a 3-D volume (two spatial axes, *x* and *y*, and an intensity axis *i*). Fractal dimension measures the degree to which image content fills the 3-D volume of the *xyi* space. The measure varies between a minimum of 0 (uniform field) and maximum of 3 (highly intricate pattern filling the *xyi* space.

*Entropy* (EN). Entropy [24] refers to the number of bits required to encode an image, and was calculated using the built-in ‘entropy’ Matlab^®^ function. Entropy values varied between 0 (highly redundant, e.g., uniform field) and 8 (highly unpredictable, randomly varying 8-bit image).

Relevant Matlab scripts are available at the Open Science Framework:

DOI 10.17605/OSF.IO/9U36Z (https://osf.io/9u36z/)

### 2.4. Fitting Image Statistics to Ratings

Pearson correlations were calculated between the ratings and the nine sets of statistics. The significance level of the correlations was adjusted for multiple tests using FDR correction [25]. Matlab’s^®^ PLSR function (plsregress) was applied to the image statistics and ratings. PLSR constructs new predictor variables (components) as linear combinations of the nine predictor variables (3 image statistics x 3 colour planes), to account for as much variance in the aesthetic ratings as possible. Image statistics were centred and scaled (converted into z-scores) prior to regression, in order to ensure that each statistic was given equal weight in the model fitting. In order to avoid over-fitting, the number of components in each fitted model was restricted to the minimum number required for the percentage of variance explained by the model to begin reaching an asymptote (i.e., components needed to reach 85% of the asymptotic value).

The VIP score that was calculated for each model component is based on the variance explained by that component. It is calculated in such a way that the average of squared VIP scores in the model equals 1, so components with a VIP score greater than 1 are more important than average. A criterion of VIP > 1.25 was used for component selection in the present analysis, because this is recommended as an appropriate value for selecting a small subset of components from highly correlated predictor variables [19].

## 3. Results

Table 1 summarises the Pearson correlations between ratings of different sub-sets of the images (in rows), and the statistical measures (columns). The left-most data column reports the number of images in each set. Each correlation value is expressed in terms of the percentage of variance shared between the ratings of the image set in the relevant row and the statistic in the relevant column (Coefficient of Determination, Cd, calculated as r^2^ × 100). The values in bold are significant at the 5% level using FDR correction.

With one exception, the PLSR models in Table 2 account for far more variance in ratings that do the individual statistics in Table 1. PLSR models account for up to 74.97% of the variance in ratings, whereas the highest Cd for any individual correlation in Table 1 is 28.47%. The single exception concerns the analyses of the entire set of images. Here, all except one of the individual statistics yielded significant correlations with higher Cd values (up to 22.49%) than a PLSR model containing four components (6.46%).

The third row in Table 1 shows correlations of individual statistics with landscape ratings. Entropy in luminance EN(L) is most highly correlated with ratings, with a Cd of 20.98%. EN(L) also yields the highest VIP score in the PLSR model (Table 2). The weighting of EN(L) in the largest components of the model is positive (as is the Pearson correlation), indicating that higher luminance entropy or unpredictability is important for higher ratings of landscapes.

For still life artworks, no individual statistics are significantly correlated with ratings. However, a three-component model explains 34% of the variance in ratings. EN(b) makes the greatest contribution to the model. Its weight in the largest components is positive, so higher entropy is again associated with higher ratings.

For portraits, although two statistics were individually correlated with ratings (SL(L) and EN(L)), the variance explained was relatively low (8.6% and 5.4% respectively) compared to a two-component model (18.6%). SL(L) made the greatest contribution to this model and was negatively weighted in the largest components of the model, so lower slope values (i.e., steeper negative slopes) were associated with higher ratings.

For nudes, luminance spectral slope is individually correlated with ratings (Cd 23.65%), and a three-component model explained 74.97% of the variance. Luminance spectral slope SL(L) was also the most important statistic for the model and was positively weighted. Thus, higher slope values (i.e., shallower negative slopes) were associated with higher ratings.

For images containing groups of people, no individual statistics were significantly correlated with ratings. A four-component model explained 11.01% of variance, with R-G entropy EN(a) making the greatest contribution to the fit. This statistic was negatively weighted in the largest model components, so lower entropy was associated with higher ratings.

For paintings of animals, no individual statistics are significantly correlated with ratings. However, a six-component model explained 46.14% of the variance in ratings, B-Y fractal dimension FD(b) making the most important contribution to the fit. This statistic was negatively weighted in the largest model components, indicating that lower FD values were associated with higher ratings.

For paintings containing buildings or other constructions, no individual statistics were significant, but a four-component model explained 54.61% of the variance in ratings. Spectral slope was most important, particularly in R-G SL(a). This statistic was negatively weighted in the largest model components, indicating that lower slope values (i.e., steeper negative slopes) were associated with higher ratings.

For abstracts, no individual statistics were significantly correlated with ratings. On the other hand, a four-component model explained 13.14% of variance, with EN(L) making the greatest contribution. It was negatively weighted, so lower luminance entropy was associated with higher ratings.

## 4. Discussion

The relatively poor fit of a single multi-component PLSR model to the entire image set, in combination with the better model fits to separate sub-sets of images, indicates that the subject matter or genre of paintings should be taken into account when considering the role of image statistics in aesthetic ratings. Different sub-sets of image statistics appear to be most important for different subjects.

**Figure 1:**
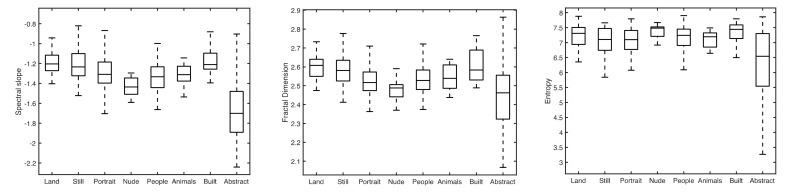
Boxplots of luminance statistics of different painting genres. Left: spectral slope. Middle: Fractal Dimension. Right: Entropy.

The diversity of the relationships between image statistics and aesthetic ratings across genres suggests that either: (i) different genres vary in their image statistics, and this variation determines which statistics are most relevant for ratings; or (ii) different statistical characteristics are relevant to ratings of different kinds of scene. In order to evaluate the first explanation, statistical analyses were performed on the image statistics for different genres. Figure 1 shows boxplots of the luminance statistics of different genres for spectral slope (left-most graph), fractal dimension (middle graph) and entropy (right-hand graph). The central line in each box marks the median value, and the bottom and top edges of the box indicate the 25th and 75th percentiles respectively. The whiskers extend to the most extreme data points not considered to be outliers. Distributions of colour plane statistics were similar to the luminance statistics shown.

Two features are most apparent in the graphs. First, the statistical distributions of all genres apart from abstracts tend to overlap, as shown by the box positions on the *y*-axes. Second, any variations that are apparent, such as in the distributions of scores for nudes and for abstracts compared to other genres, are fairly consistent across the three statistics. One-factor ANOVAs were computed to test the effect of genre on image statistics, and found a significant main effect of genre for all three statistics (SL: F (7, 463) = 35.72, *p* < 0.0001; FD: F (7, 463) = 11.17, *p* < 0.0001; EN: F (7, 463) = 13.02, *p* < 0.0001). However, within each visual statistic, multiple comparisons of all 28 possible pairings of the eight genres indicated that these significant main effects were due almost entirely to the abstract genre being different from other genres (as indicated by inspection of Figure 1): In the case of SL, six of the eight significant paired comparisons involved abstracts (the other two were land vs. people and land vs. nudes); in the case of FD, five of the six significant paired comparisons involved the abstract genre (the other one was land vs. portraits); in the case of EN, all of the seven significant paired comparisons involved the abstract genre.

Thus, there is little support for the suggestion that genre-by-genre variations in statistical properties are responsible for the variations in the relative importance of different statistics in the best-fitting models across different genres. The alternative explanation is that different statistical characteristics are relevant to ratings of different kinds of scene, at least for some of the genres. Not only are different image statistics important for ratings in different genres, but the direction of the association also varies as indicated in the description of the results. In particular, higher entropy is preferred in paintings of landscapes and still life subjects, but lower entropy is preferred in paintings of people and abstracts. Steeper spectral slopes are preferred in portraits, but shallower slopes are preferred in nudes. The challenge then is to understand why different particular statistical relationships best capture the attributes of different subjects that are most relevant to aesthetic ratings.

It is well established that different neural sub-systems are involved in the processing of faces (Fusiform Face Area FFA [26]), bodies (Extrastriate Body Area EBA [27]) large scenes (Parahippocampal Place Area PPA [28]), and objects (Lateral Occipital Complex LOC [29]). Moreover, a recent meta-analysis of 47 fMRI experiments on visual aesthetic experiences [30] found that different cortical areas are involved, dependent on subject matter. The different areas associated with aesthetic experiences of portraits, real-world scenes, abstracts and body sculptures corresponded with those that had been identified previously (e.g., FFA, PPA). The importance of subject matter could indicate that these different neural sub-systems diverge in terms of the image statistics that influence their processing efficiency and output.

According to the VIP scores, photometric statistics (based on spectral slope) are most important for nudes, and are also important for faces, particularly in terms of luminance. As a photometric measure, spectral slope may capture subtle variations in light and shade to model spatial form and lighting effects with high precision (a technique developed by Leonardo da Vinci, known as chiaroscuro). The different directions of the relationships between spectral slope and ratings of portraits versus nudes indicate that there are subtle but aesthetically significant differences in the way the human form is modelled in these two genres. Colour photometry is important for paintings that contain urban scenes, building and interiors, and this could be related to the use of light and shade (variation in colour tone and shading). On the other hand, for paintings that contain animals or still life subjects, geometric statistics are most important, particularly in the B-Y colour axis. So it seems that in these subjects it is the complexity and degree of disorder of the patterns that is most relevant to ratings. Paintings in these genres can contain all kinds of objects such as flowers, fruit, vegetables, fish, poultry, furniture, pottery, drapery, and so on. The objects involved, and their depiction, are often chosen for the sake of their shape, colour, texture and composition. On the basis of the best-fitting PLSR models, it seems that pattern complexity and orderliness are most relevant for aesthetic appeal, rather than photometric qualities. Higher degrees of unpredictability are liked most in landscapes and still life paintings, but not in abstract paintings. Yet, of course, these interpretations are rather post hoc. For some genres in the image set, the best statistical models account for less than a third of the variance in ratings, so other factors than image statistics are clearly of primary importance. The semantic content and meaning of paintings must exert an important influence on aesthetic responses that cannot be captured by image statistics.

## Figures and Tables

**Table 1:** Summary of correlations between image statistics (columns) and aesthetic ratings of paintings in different genres (rows).

Genre	*n Image*	*SL(L)*	*SL(a)*	*SL(b)*	*FD(L)*	*FD(a)*	*FD(b)*	*EN(L)*	*EN(a)*	*EN(b)*
***All art***	476	**22.49**	**0.92**	0	**6.69**	**15.22**	**15.12**	**17.04**	**12.93**	**19.04**
***Abstract***	80	1.03	1.58	0.36	0.02	0.03	0	0.24	5.67	5.04
***Landscape***	51	1.38	2.33	0.58	1.12	2.46	0.03	**20.98**	1.1	**16.72**
***People***	131	3.27	0.21	0.74	1.18	1.72	0.76	0.02	2.37	0.71
***Still Life***	29	5.41	0.02	1.56	8.49	6.83	0.23	1.06	2.88	3.25
***Portrait***	133	**8.56**	0.18	1.89	1.45	3.44	1.48	**5.4**	0.1	2.78
***Nude***	14	23.65	1.28	13.69	6.99	0.02	2.48	0.1	0.92	3.58
***Animals***	17	25.6	6.77	0	13.35	1.32	1.62	16.85	0.05	0.7
***Built***	16	0.03	28.47	27.63	0	0.01	0.08	0.07	3.9	2.4

Correlations are reported as Coefficients of Determination, Cd, calculated as (r^2^ × 100). Values in bold are significant at the 5% level or higher, after adjustment for False Discovery Rate.

**Table 2:** Summary of best-fitting PLSR models applied to paintings in different genres (rows).

Genre	*n Comp*	*Cd*	*SL(L)*	*SL(a)*	*SL(b)*	*FD(L)*	*FD(a)*	*FD(b)*	*EN(L)*	*EN(a)*	*EN(b)*
***All art***	4	6.46	1.02	**1.39**	0.75	0.63	0.87	0.64	0.42	**1.65**	1
***Abstract***	4	13.14	0.75	0.74	**1.37**	1.01	0.68	0.45	**1.53**	0.89	1.09
***Landscape***	2	22.06	0.51	**1.45**	1.17	0.55	0.67	0.61	**1.51**	0.23	**1.34**
***People***	4	11.01	0.28	0.59	1.12	0.69	1.02	1.07	0.7	**1.85**	0.85
***Still Life***	3	33.96	1.16	0.55	0.55	**1.36**	0.45	**1.3**	0.58	0.91	**1.46**
***Portrait***	2	18.56	**1.66**	0.76	0.46	0.52	1.09	0.72	**1.32**	0.86	1
***Nude***	3	74.97	**2.2**	0.17	1.1	0.61	0.26	0.99	0.55	0.96	0.55
***Animals***	6	46.14	0.9	**1.33**	0.62	1.06	1.13	**1.49**	0.6	0.64	0.8
***Built***	4	54.61	0.7	**1.68**	**1.59**	0.66	0.51	0.64	0.97	0.78	0.69

‘n Comp’ is the number of components in the model; ‘Cd’ is the Coefficient of Determination for the model fit, defined as in Table 1; cell entries are VIP scores for the image statistic in the corresponding column (values in bold exceed the criterion value of 1.25).
