# Correction: Mather, G. Aesthetic Image Statistics Vary with Artistic Genre. *Vision* **2020**, *4*, 10

**DOI:** 10.3390/vision5020025

**Published:** 2021-05-24

**Authors:** George Mather

**Affiliations:** School of Psychology, University of Lincoln, Lincoln LN6 7AY, UK; gmather@lincoln.ac.uk

The author wishes to make the following corrections to the paper [1]:

During additional data analyses after publication of the manuscript, the author found an error in the Matlab script that calculated the coefficients of determination (Cd) between individual image statistics and aesthetic ratings of the image sets. Due to this error, a small subset of the images in each set was excluded from the calculation. The correct values for Table 1 are shown below.

Correlations are reported as coefficients of determination, Cd, calculated as (r^2^ × 100). Values in bold are significant at the 5% level or higher, after adjustment for the false discovery rate.

References to the relevant Cd values in the “3. Results” section should be changed to reflect the corrected values. For example, the correct Cd values for the entire set of images (top row in the table) are much lower than those originally reported. For instance, for *SL(L)*, the correct value is 0.16, not 22.49 as reported.

The author would like to apologise for any inconvenience caused to readers by these changes. The errors do not significantly change the conclusions of the paper. The corrected Cd values are almost all lower than those originally reported, reinforcing the points already made that (1) individual image statistics generally explain relatively little of the variance in aesthetic ratings; and (2) multi-component partial least squares regression models account for far more variance than single-component correlations. 

## Figures and Tables

**Table 1:** Summary of correlations between image statistics (columns) and aesthetic ratings of paintings in different genres (rows).

Genre.	*n Image*	*SL(L)*	*SL(a)*	*SL(b)*	*FD(L)*	*FD(a)*	*FD(b)*	*EN(L)*	*EN(a)*	*EN(b)*
All art	476	0.16	1.55	0.01	0.3	0.56	0.31	0.09	**1.47**	0.05
Abstract	80	0	0.32	3.63	0.3	0.22	0.47	0.35	2.4	3.17
Landscape	51	0.84	7.54	5.08	0.38	1.43	0	9.26	0.02	7
People	131	0.03	0.52	0.59	0.3	0.25	0	0.03	5.05	0.86
Still Life	29	5.15	0.21	0.59	5.67	0.16	2.59	1.18	0.16	7.01
Portrait	133	**5.22**	0.5	0.73	0.11	3.94	1.84	**5.72**	0	3.65
Nude	14	45.67	0.01	10.78	1.91	0.12	7.16	0.32	7.55	2.43
Animals	17	4.41	7.12	2.09	11.4	5.71	8.57	0.57	0.79	0.48
Built	16	5.9	32.26	32.1	2.28	0.09	0.2	0.75	4.25	1.8
